# Advances in Electrofusion Welding Technology for Polymeric Pipelines: From Process Optimization to Mechanism-Driven Control

**DOI:** 10.3390/polym18111402

**Published:** 2026-06-05

**Authors:** Bingyuan Hong, Zhongjian Sun, Zenan Wu, Yu Meng, Zhiwei Chen, Xianlei Chen, Weiqiang Wang, Daiwei Liu

**Affiliations:** 1 National & Local Joint Engineering Research Center of Harbor Oil & Gas Storage and Transportation Technology, Zhejiang Key Laboratory of Petrochemical Environmental Pollution Control, Zhejiang Key Laboratory of Pollution Control for Port-Petrochemical Industry, School of Petrochemical Engineering & Environment, Zhejiang Ocean University, Zhoushan 316022, China; 2 Zhoushan Institute of Calibration and Testing for Quality and Technology Supervision, Zhoushan 316000, China; 3 Zhejiang HengAnTai Petroleum Engineering Co., Ltd., Zhoushan 316013, China

**Keywords:** polymeric pipelines, electrofusion welding, temperature field, residual stress, welding defects

## Abstract

With the rapid development of clean and low-carbon energy systems, non-metallic pipelines have become increasingly important in urban gas distribution, water supply, and emerging energy-transport applications, including hydrogen service. As a critical joining technology that governs system integrity and long-term operational safety, electrofusion welding requires a comprehensive and mechanism-oriented understanding beyond empirical process control. In this study, a review is conducted on research published over the past decade in the field of electrofusion welding of non-metallic pipelines, with emphasis on fundamental technical issues including the formation and evolution of temperature fields, characteristics of the molten fusion zone and defect development, and thermo-mechanical coupling with residual stress generation. Based on a synthesis of the literature, the review clarifies the global research landscape, core research communities, and underlying knowledge structure. The results indicate a clear transition of the field from empirically driven parameter optimization toward a mechanism-based and process-controllable paradigm centered on temperature field evolution, fusion zone development, and thermo-mechanical behavior. Current research hotspots converge on HDPE material adaptability, welding process regulation, and the long-term reliability of welded joints. Building on these insights, future research directions are discussed, including mechanism-driven process design, intelligent defect identification based on multi-source data, and full-life reliability assessment under service conditions. This review provides a theoretical framework to support process optimization and engineering application of electrofusion welding in non-metallic pipeline systems.

## 1. Introduction

Driven by the global transition toward clean and low-carbon energy systems, the application of polymeric pipelines has expanded rapidly in urban gas distribution, water supply, and emerging energy-transport infrastructures. Compared with conventional metallic pipelines, polymer-based pipelines—such as polyethylene (PE) and polyamide (PA)—offer distinct advantages, including corrosion resistance, low density, and ease of installation. Among available joining technologies, electrofusion welding has become one of the most widely adopted methods for polymeric pipeline systems due to its operational convenience and adaptability to complex field conditions. Existing engineering practices and experimental studies have demonstrated that welded joints represent mechanically and structurally complex regions within pipeline systems, and their performance plays a critical role in determining overall system reliability.

With the rapid development of clean and low-carbon energy systems, polymeric pipeline systems have been increasingly applied in gas transportation, water supply and drainage [1], chemical engineering, and emerging hydrogen transportation fields [2]. Owing to its excellent sealing performance, high joint reliability, and strong adaptability to complex on-site construction environments, electrofusion welding has become one of the most widely adopted joining technologies at present [3,4,5]. Compared with traditional mechanical joining methods, electrofusion welding enables more stable interfacial bonding and effectively reduces the risk of leakage under long-distance buried service conditions. In practical engineering applications, electrofusion welding is particularly suitable for complex scenarios such as pipeline repair, branch connection, and construction in confined spaces, thereby exhibiting high engineering adaptability and relatively stable welding quality. In recent years, driven by the increasing demands for infrastructure safety, intelligent pipeline networks, and low-carbon energy transportation, the industrial application value of electrofusion welding technology has been further enhanced. In particular, in hydrogen-blended and pure hydrogen transportation systems, the long-term integrity and reliability of polymeric welded joints have become critical engineering concerns [6], which has consequently promoted continuous research in this field regarding mechanism analysis, defect control, and intelligent quality assessment [7,8,9].

Existing research on electrofusion welding of polymeric pipelines has been carried out from multiple perspectives, including heat transfer behavior, molten zone formation, material rheology, interfacial diffusion, and crystallization evolution [10]. These studies have collectively elucidated the complex coupling relationships among the temperature field, stress field, and microstructural evolution during the welding process. For instance, Lai [11] demonstrated that non-uniform temperature distribution during electrofusion welding not only governs the thickness and morphology of the effective molten zone, but also induces residual stress accumulation within the joint by influencing cooling-induced shrinkage and structural constraints, thereby exerting a significant impact on both the mechanical performance and long-term service behavior of welded joints.

**Figure 1:**
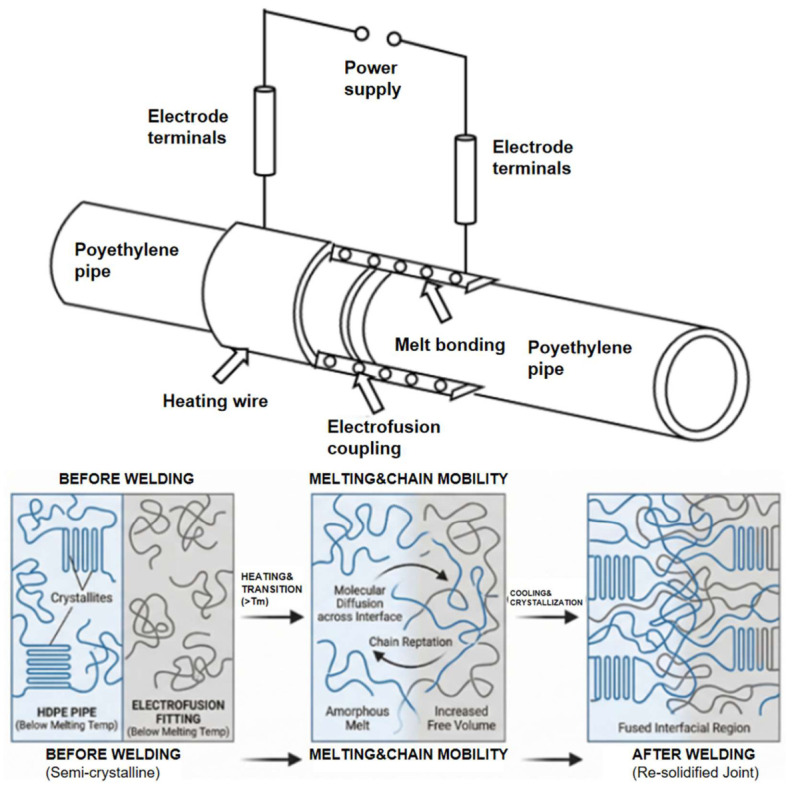
Material adaptability of HDPE for electrofusion welding and interfacial fusion mechanism.

As illustrated in Figure 1, the formation mechanisms of typical welding defects—such as incomplete fusion, porosity, localized overheating, and weak interfacial bonding—have been extensively investigated. Previous studies have attributed these defects to fluctuations in process parameters [12], as well as variations in material batches and uncertainties associated with on-site construction conditions [13].

Although significant progress has been achieved in recent years in the field of electrofusion welding of polymeric pipelines, several important limitations still exist from a holistic perspective. Most existing studies and review articles primarily focus on individual aspects such as welding parameter optimization, joint mechanical performance, or defect characterization, while lacking systematic analysis of the intrinsic relationships among temperature field evolution, molten zone formation, defect development, residual stress generation, and long-term reliability [14]. In addition, although advanced approaches such as numerical simulation, thermo-mechanical coupling analysis, microstructural characterization, and intelligent monitoring have gradually been introduced into this field in recent years, these emerging mechanism-driven research directions have not yet been integrated into a unified analytical framework. Meanwhile, most existing reviews are based on conventional narrative summaries and rarely combine bibliometric analysis with technical reviews, making it difficult to quantitatively reveal the evolution of research hotspots, collaboration networks, and future technological development trends within this field.

Among the currently used polymeric pipeline materials, high-density polyethylene (HDPE) has become one of the most widely applied materials in gas distribution, water supply systems, and industrial fluid transportation owing to its excellent overall performance. Compared with alternative materials such as PVC, PP, and PA, HDPE possesses relatively high mechanical strength, excellent crack resistance, good toughness, and outstanding corrosion resistance [15]. In addition, HDPE exhibits favorable thermoplastic processing characteristics, allowing sufficient melting, molecular chain diffusion, and interfacial entanglement during electrofusion welding, thereby facilitating the formation of highly reliable welded joints. Furthermore, from an economic perspective, HDPE offers advantages including moderate raw material cost, high installation efficiency, low maintenance requirements, and long service life, demonstrating favorable life-cycle economic performance. For these reasons, HDPE is selected in this study as the representative material system for detailed discussion.

Therefore, unlike previous review studies that mainly summarize isolated technical issues, the present work integrates bibliometric analysis with a mechanism-oriented technical review to establish a systematic analytical framework centered on the progression of “temperature field evolution–fusion zone formation–welding defects–residual stress development–long-term reliability,” thereby enabling systematic linkage among different research levels. Meanwhile, this review particularly emphasizes the ongoing transition of the field from conventional empirical parameter optimization toward mechanism-driven process control and intelligent quality assessment. Emerging research directions, including intelligent defect diagnosis, multi-source data fusion, and full-life reliability evaluation, are also further discussed. By combining mechanistic analysis with quantitative investigation of literature structures, this study aims to provide a more systematic research perspective for process optimization, joint reliability enhancement, future research, and engineering applications of electrofusion welding for polymeric pipelines.

The main contributions of this study are summarized as follows:

(a) This study reconstructs the intellectual evolution of electrofusion welding in polymeric pipelines through bibliometric analysis of publications from 2015 to 2025. The results reveal a transition from empirical process optimization toward a mechanism-driven research framework emphasizing temperature field evolution, molten zone formation, and thermo-mechanical coupling.

(b) The global research landscape and collaboration networks are systematically analyzed, identifying major contributors and dominant research clusters. Core research themes are mainly HDPE materials, welding process control, and joint performance evaluation, reflecting the highly international and interdisciplinary nature of the field.

(c) A technical framework centered on the progression of “temperature field–molten zone–defects–residual stress–reliability” is established. This framework links fundamental welding mechanisms with joint structural response and long-term service performance.

(d) Future research is expected to focus on mechanism-driven process design, intelligent defect identification based on multi-source data, and full-lifecycle reliability assessment under thermo-mechanical coupling. These directions will support safer and more reliable polymeric pipeline systems for emerging energy transportation applications.

## 2. Bibliometric Analysis of Polymeric Pipeline Welding Research

### 2.1. Data Collection and Method

This study employs bibliometric approaches, including co-word analysis, clustering analysis, and knowledge mapping, to systematically investigate research hotspots and developmental trends in the field of electrofusion welding of polymeric pipelines. The data were retrieved from the Web of Science database, with search terms primarily centered on “welding,” “electrofusion welding,” “heat fusion welding,” and “polymeric pipelines,” thereby ensuring comprehensive coverage of the core research topics in this domain. The time span was limited to the most recent decade (2015–2025), which not only ensured the timeliness of the analysis but also captured the overall developmental trajectory and latest advances in electrofusion welding technologies for polymeric pipelines.

The bibliometric analysis follows a widely adopted five-stage framework, comprising research design, data collection, data analysis, data visualization, and interpretation [16]. In practice, the selected publications that met the inclusion criteria were first exported as structured text data and subsequently imported into specialized bibliometric software, including VOSviewer 1.6.20 and CiteSpace 6.2.R4. Through algorithm-driven computations, these tools enable the construction of visual knowledge maps and statistical analyses. By generating knowledge graphs, the study examines the research landscape and its evolution from multiple dimensions, including author collaboration networks, institutional distribution, keyword co-occurrence, and burst term detection. Specifically, VOSviewer is primarily used for network visualization and relational mapping, whereas CiteSpace focuses on data preprocessing and advanced bibliometric analysis. The combined use of these tools facilitates the transformation of raw bibliographic data into an interpretable knowledge structure.

### 2.2. Analysis of Bibliometric Results

**Figure 2:**
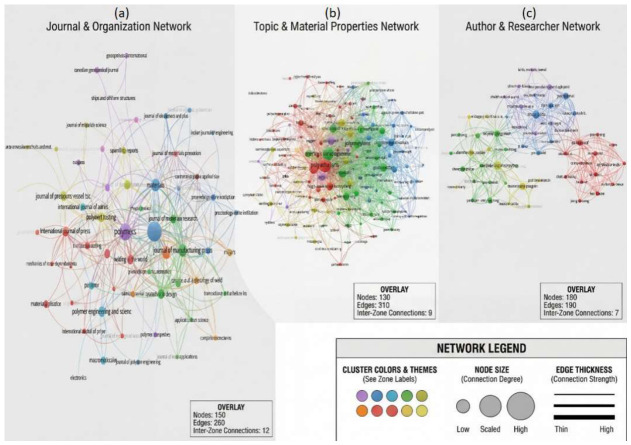
VOSviewer-based symbiotic network analysis. Different colored regions represent different clustering results. The connecting lines indicate that the information is interrelated. The size of each node represents the frequency of occurrence of information: (**a**) Journal clustering analysis; (**b**) Keyword clustering analysis; (**c**) Author co-occurrence clustering analysis.

From the perspective of polymeric pipeline welding, Figure 2a reveals a knowledge structure centered on polymer materials, manufacturing processes, and performance evaluation. Journals such as *Polymers*, *Journal of Manufacturing Processes*, *Journal of Materials Research*, and *Polymer Engineering and Science* occupy central positions within the network, indicating that the foundational research on polymeric pipeline welding (e.g., electrofusion and heat fusion welding of HDPE) is primarily rooted in polymer science and processing mechanisms.

Meanwhile, journals such as *Welding Journal* and *Welding in the World* form a closely connected cluster, reflecting a strong focus on welding parameter control, joint mechanical performance, quality assessment, and failure analysis. In addition, the involvement of journals such as *Composites Part A*, *Materials & Design*, and *Composite Structures* suggests that the field is progressively extending toward composite materials and high-performance pipeline structures.

Furthermore, interdisciplinary journals including *Scientific Reports*, *Sensors*, and *Applied Surface Science* highlight the growing role of microstructural characterization, surface engineering, and monitoring technologies in supporting the reliability assessment of welded joints. Overall, this network illustrates that research on polymeric pipeline welding is strongly application-driven, characterized by a deep integration of material mechanisms and manufacturing processes.

Figure 2b provides a clear representation of the research landscape in polymeric pipeline welding. Polyethylene—particularly high-density polyethylene (HDPE)—emerges as the dominant material, with studies on its welding technologies and performance forming the core of the field. The prevailing joining techniques are primarily butt fusion and electrofusion welding, while emerging methods such as friction stir welding and laser welding are also being actively explored.

The central research objective is to enhance the mechanical performance of welded joints, including strength and tensile properties, through the optimization of processing parameters. At the same time, considerable attention has been devoted to predicting the long-term service performance of pipelines by analyzing failure mechanisms, fatigue behavior, microstructural morphology, and crystallinity.

Furthermore, the knowledge map highlights several emerging research directions. These include the incorporation of nanomaterials, such as graphene oxide and carbon nanotubes, for pipeline modification, as well as the integration of advanced technologies—such as additive manufacturing and X-ray inspection—to further advance the field.

Figure 2c clearly illustrates the global landscape of scientific collaboration within the field of polymeric pipeline welding. Several core collaborative clusters can be identified, represented by distinct colors such as red, blue, green, and yellow. The red cluster is primarily composed of Chinese researchers, including Yan, Zhang, Wang, and Xiao, and focuses on polyethylene pipeline welding and performance optimization.

The blue cluster, represented by Shen, Yifu and Pal, and Kamal, connects researchers from multiple countries, including China, India, and Malaysia. This cluster emphasizes process parameter optimization and joint mechanical performance, and serves as one of the most prominent bridges for international collaboration. In contrast, the green and yellow clusters, centered around Deveci, Suleyman and Iurzhenko, and Maksym, represent research groups from Europe and Central Asia, with a particular focus on welding failure analysis and material modification.

Although some cross-regional linkages exist among these clusters, the overall depth of international collaboration remains limited, indicating significant potential for further strengthening global research integration. Notably, the research themes represented by these clusters are highly consistent with the core topics and emerging frontiers of the field.

**Figure 3:**
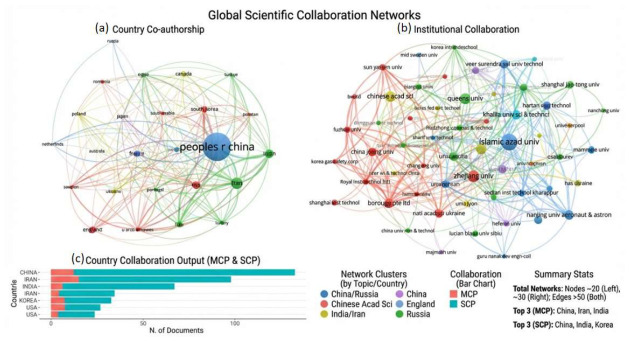
VOSviewer-based symbiotic network analysis. Different colored regions represent different clustering results. The connecting lines indicate that the information is interrelated. The size of each node represents the frequency of occurrence of information: (**a**) Country clustering analysis (**b**) Institution clustering analysis; (**c**) Distribution of publications across different countries in the field of polymeric pipeline welding.

Figure 3c illustrates the distribution of publications across different countries in this research field. The color coding distinguishes between different types of publications. Specifically, blue (SCP) denotes *Single Country Publications*, referring to publications in which all authors are affiliated with the same country, whereas red (MCP) represents *Multiple Country Publications*, indicating studies conducted through international collaboration involving authors from multiple countries.

This classification provides valuable insights into the relative contributions of different countries to both domestically driven research and internationally collaborative outputs, thereby reflecting the degree of globalization and cooperation within the field.

From the perspective of polymeric pipeline welding, Figure 3a clearly depicts the global research landscape of this field. China (People’s Republic of China) appears as the largest node, highlighting its dominant role in both research output and international collaboration. Countries such as India and Iran, represented within the green cluster, form important regional collaboration networks, while nations including the United States, the United Kingdom, and South Korea—grouped in the red cluster—along with France and Russia, represented by blue and light-blue nodes, also constitute active research contributors.

The dense interconnections among countries indicate a high level of international collaboration, with China serving as a central hub linking research partners worldwide. This pattern underscores the inherently global and collaborative nature of research on polymeric pipeline welding and related core technologies. Moreover, the presence of distinct color clusters suggests subtle regional differences in research focus and technological priorities across countries.

Figure 3b provides a clear depiction of the distribution of research capacity and the collaboration network within this field. A highly active research cluster, represented in red, is centered around major Chinese institutions, including Zhejiang University, the Chinese Academy of Sciences, and Shanghai Jiao Tong University. The close involvement of industrial entities, such as BOROUGE PTE LTD, not only highlights China’s leading position in this domain but also underscores the strong integration of academia, industry, and research.

At the same time, institutions from other regions contribute significantly to the global network. These include Middle Eastern universities such as Islamic Azad University and Khalifa University of Science and Technology, Eurasian research nodes such as the Korea Institute of Industrial Technology and the National Academy of Sciences of Ukraine, as well as universities from Europe and North America, including Queen’s University and KTH Royal Institute of Technology.

Together, these institutions form a cross-regional and highly interconnected international collaboration network, reflecting a comprehensive cooperative chain spanning fundamental research, technological development, and engineering applications in the field of polymeric pipeline welding.

## 3. Advancements in Polymeric Pipeline Welding

Building upon the preceding bibliometric analysis, this study systematically elucidates the research landscape, knowledge structure, and global collaboration patterns in the field of polymeric pipeline welding. Three major research hotspots are identified, namely: (a) process control issues centered on temperature field evolution and the formation mechanisms of the effective molten zone during welding; (b) the formation and evolution of welding defects and residual stresses; and (c) the assessment of structural integrity and long-term reliability of welded joints under service conditions.

On this basis, the present work further focuses on the state-of-the-art research in electrofusion welding of polymeric pipelines. The existing studies are organized into several interrelated and progressively advancing technical layers, thereby establishing a systematic analytical framework for the field.

The following sections are structured according to the evolutionary pathway of technical understanding in electrofusion welding of polymeric pipelines. First, at the material and weldability level, the physical and thermal properties of typical polymer materials, such as high-density polyethylene (HDPE), are examined to establish the material basis for achieving reliable joints under electrofusion conditions. Second, at the process parameter level, the effects of key parameters—such as current, voltage, and heating time—on joint formation quality and mechanical performance are systematically analyzed.

Building upon this, the focus shifts to the welding process evolution level, where the formation mechanisms of non-uniform temperature fields and effective molten zones are investigated, enabling the establishment of intrinsic relationships between welding processes and defect evolution. Subsequently, at the structural response and service behavior level, thermo-mechanical coupling and residual stress analyses are introduced to evaluate internal structural responses and potential failure risks within welded joints. Finally, at the engineering application and long-term reliability level, a multi-scale, mechanism-informed perspective is adopted to explore pathways for enhancing the long-term safety and reliability of polymeric pipeline systems through process optimization and advanced control strategies.

**Figure 4:**
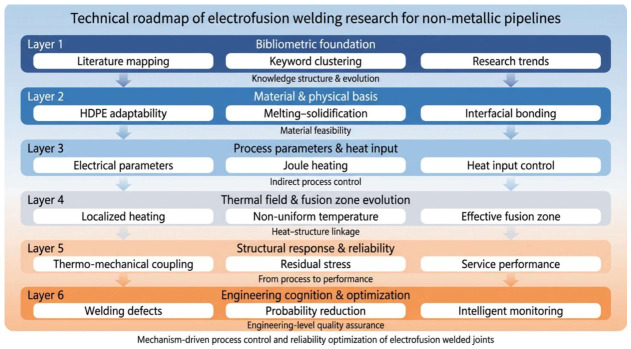
Technical roadmap of the study created by the authors.

In summary, as shown in Figure 4, the bibliometric analysis not only identifies three central research hotspots—namely interfacial compatibility in welding, optimization of process parameters, and the long-term reliability of welded joints—but also provides a structured roadmap for further technological investigation.

Building upon these insights, the following sections will present a systematic and in-depth discussion of the fundamental principles of electrofusion welding, the types of defects induced by anomalies in molten zone formation, methods for evaluating joint performance and residual stresses, as well as the key technical challenges and bottlenecks encountered in engineering applications. Furthermore, future research directions will be explored, with the aim of providing both theoretical guidance and practical support for the innovative development of welding technologies for polymeric hydrogen transportation pipelines.

### 3.1. Formation and Evolution Characteristics of the Temperature Field in Electrofusion Welding

Studies such as Mansouri [17] have demonstrated that the performance of electrofusion welded joints is governed not only by externally imposed process parameters, but also by the evolution of internal thermal processes during welding. Unlike conventional bulk heating methods, electrofusion welding relies on embedded resistive heating elements to achieve localized volumetric heating, which inevitably leads to the formation of non-uniform temperature fields within the welding zone [18].

#### 3.1.1. Localized Volumetric Heating Mechanism in Electrofusion Welding

The heat input mechanism of electrofusion welding is fundamentally different from that of conventional heat fusion welding. Unlike external heating approaches, in which the joint interface is heated uniformly by an external heat source, electrofusion welding utilizes embedded resistive heating elements (typically resistance wires) to generate heat directly within the joint, thereby achieving localized volumetric heating of the welding region. Avrigean [19] demonstrated that the underlying physical mechanism is governed by Joule heating, whereby the electric current passing through the conductive element generates heat that is directly deposited within the material. As a result, thermal energy initially accumulates in the weld zone and subsequently diffuses into the surrounding material.

Building upon the understanding that internal Joule heating serves as the primary heat source in electrofusion welding, subsequent studies have focused on the spatial distribution of heat input and its influence on temperature field evolution. Guo [20] reported that the heat source in electrofusion welding is inherently non-uniform, being concentrated around the heating wire and its adjacent regions, and progressively diffusing in both radial and axial directions over time. This localized volumetric heating characteristic results in a pronounced non-uniform temperature field within the joint, where the interfacial region experiences rapid temperature rise, while regions farther from the heating element remain comparatively cooler.

**Figure 5:**
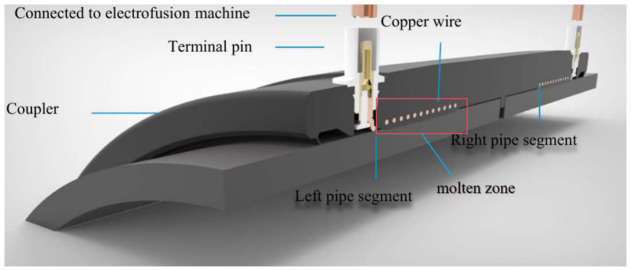
Schematic illustration of the fusion zone formation in electrofusion welding of polymer pipelines [17].

Furthermore, Obedan [21] employed numerical heat transfer modeling to demonstrate that the coupled effects of material thermophysical properties, heating parameters, and geometric conditions significantly influence heat input and thermal diffusion behavior, thereby governing the formation and evolution of the molten zone. Localized Joule heating from the embedded resistive element generates a non-uniform temperature field, driving interfacial melting and outward expansion of the fusion zone. The spatial distribution of the molten region is illustrated in Figure 5.

In addition, from the perspective of experimental thermal analysis, techniques such as differential scanning calorimetry (DSC) have demonstrated that polyethylene undergoes significant crystallization changes and heat diffusion processes during welding [22]. These thermal responses are influenced not only by the heating rate but also strongly depend on the spatial distribution of heat released by localized heat sources. Savu [23] emphasized that the localized volumetric heating mechanism in electrofusion welding differs from conventional welding not merely in terms of energy input mode, but also in the resulting microstructural response of the material.

Therefore, compared with traditional welding methods, the localized volumetric heating mechanism of electrofusion welding exhibits three defining characteristics. First, the heat source is located within the joint, resulting in heat transfer from the interior toward the surrounding material. Second, due to the inherently low thermal conductivity of polymer materials, heat cannot be rapidly or uniformly distributed, leading to pronounced temperature non-uniformity. Third, the formation of the molten zone is governed by the coupled interaction between localized heat input and the thermal response of the material.

These characteristics fundamentally determine the evolution of the temperature field during the welding process and the resulting microstructural features of the weld, thereby providing a mechanistic foundation for subsequent analyses of temperature field evolution, molten zone formation, and the performance of welded joints.

#### 3.1.2. Spatial Distribution Characteristics of Non-Uniform Temperature Fields

During electrofusion welding, the localized release of heat from embedded resistive elements leads to a pronounced spatial non-uniformity in the temperature field within the welding region [24]. Unlike conventional heat fusion welding, where the entire joint interface is heated more uniformly, the temperature field in electrofusion welding typically exhibits an axisymmetric distribution. Regions in close proximity to the heating wire rapidly reach the melting temperature, whereas areas farther from the heat source remain at comparatively lower temperatures due to limited heat conduction.

**Figure 6:**
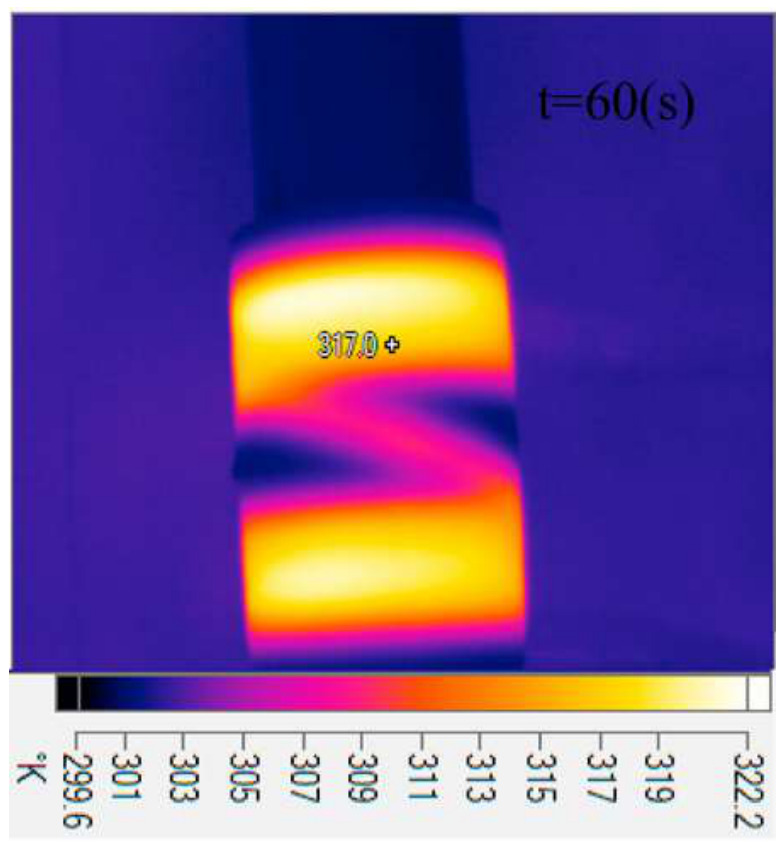
Thermal image of the joint outer surface during the electrofusion heating process [17].

Under such conditions, both the magnitude and direction of the temperature gradient are governed not only by the geometric configuration of the heating element, but also by factors such as pipe wall thickness, material thermophysical properties, and modes of heat transfer. These characteristics have been systematically validated through numerical simulations and experimental analyses, as reported by Zheng [25]. Infrared thermography reveals a pronounced spatially non-uniform temperature distribution on the joint outer surface during the heating stage, as shown in Figure 6, reflecting localized heat generation and transient heat diffusion.

In studies of temperature field modeling, the spatial distribution of temperature during electrofusion welding exhibits pronounced temporal and spatial variations. Zheng [25] developed a one-dimensional transient heat conduction model to simulate the evolution of the temperature field during electrofusion welding. The results demonstrate that heat propagates progressively from the heating wire into the surrounding pipe material, with significant differences in both peak temperature and time-to-peak at different spatial locations. Such spatial non-uniformity directly influences the formation quality of the effective molten zone, thereby governing the ultimate mechanical performance of the welded joint.

To further investigate the influencing factors of temperature distribution, researchers have conducted combined experimental and numerical studies. Stetiu [18] examined the temperature evolution at the center and edge of fittings with different dimensions during the heating process. The findings indicate that smaller-diameter fittings reach peak temperature more rapidly at the center, whereas larger-diameter joints exhibit a more gradual temperature distribution, highlighting the significant influence of geometric scale on temperature field non-uniformity. In addition, Chebbo [26] reported that variations in welding time and input conditions lead to differences in both the spatial temperature gradient and the location of peak temperature.

Collectively, these studies reveal several characteristic features of the temperature field in electrofusion welding. First, a pronounced radial temperature gradient exists, with a high-temperature zone forming rapidly near the heating element and gradually decreasing toward the outer wall. Second, due to the axisymmetric arrangement of the heating wire, the overall temperature field exhibits an axisymmetric distribution. Third, factors such as welding time, input power, and environmental conditions significantly affect the magnitude of temperature gradients and the rate of heat diffusion, thereby determining whether different regions can reach and sustain the effective melting temperature for an adequate duration.

Understanding these spatial distribution characteristics is essential for accurately defining the effective molten zone, analyzing microstructural evolution, and evaluating the performance of welded joints.

#### 3.1.3. Heating Rate Differences Between Fittings and Pipes and the Formation of Temperature Gradients

During electrofusion welding, differences in geometry, thickness, and heat transfer pathways between the fitting and the pipe result in markedly different heating rates during the welding process, leading to the formation of complex spatial temperature gradients. The embedded resistive heating element initially generates heat within the inner wall of the fitting, which subsequently propagates toward the pipe. As a consequence, the fitting region reaches the melting temperature more rapidly than the adjacent pipe material. This non-uniform thermal response constitutes a fundamental basis for weld formation and the subsequent evolution of joint properties [17].

**Figure 7:**
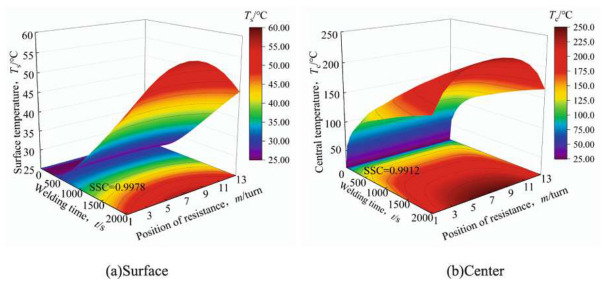
Temperature field distribution at the surface and center of the joint under standard welding conditions [27].

Early modeling studies have substantiated this phenomenon. For example, Zheng [25] developed a predictive model of the electrofusion welding temperature field, demonstrating that temperatures at the fitting–pipe interface are significantly higher than those in pipe regions farther from the heat source. Moreover, both the location of the temperature peak and the time required to reach it vary considerably due to structural differences, resulting in a pronounced temperature gradient extending from the fitting toward the pipe (shown in Figure 7).

This difference in heating rate primarily arises from disparities in thermophysical properties and geometric configurations between the fitting and the pipe. Typically, the fitting contains embedded resistance wires and exhibits a thickness distribution distinct from that of the straight pipe, resulting in differences in heat capacity, thermal conductivity, and heat exchange with the surrounding environment. Consequently, the pathways and rates of heat accumulation, conduction, and dissipation within the fitting differ significantly from those in the pipe [12]. Fujikake [28] employed thermo-mechanically coupled finite element analysis to elucidate this structurally induced spatial temperature distribution. The results indicate that the temperature within the fitting increases rapidly during heating, whereas heat encounters greater thermal resistance when diffusing into the pipe, leading to a comparatively slower thermal response in the pipe material.

Beyond structural differences in heat conduction, the intrinsic thermal response of the material also plays a crucial role in determining heating rates. Avrigean [29] conducted combined experimental and numerical analyses of temperature distributions in polyethylene joints. The study revealed that temperature-dependent variations in heat capacity and thermal diffusivity lead to more pronounced heat accumulation in the fitting region, while heat conduction in the pipe remains relatively limited. This disparity in thermal transport characteristics further amplifies the formation of localized temperature gradients.

Based on these findings, several consistent patterns can be identified. First, during the initial stage of welding, the fitting typically heats up more rapidly than the pipe, as the internal heat source (resistance wire) directly generates heat within the fitting, whereas the pipe receives heat primarily through conduction. Second, due to the inherently low thermal conductivity of polyethylene, heat cannot be uniformly distributed, and structural differences promote the rapid development of temperature gradients between the fitting and the pipe. Third, the coupled effects of environmental conditions, welding parameters, and material thermophysical properties further influence the relative heating rates and the magnitude of temperature gradients [30]. Notably, these gradients are not static but evolve dynamically over time as heat transfer progresses.

Understanding these differences in heating behavior is essential for accurately defining the effective molten zone, analyzing temperature gradient evolution, and ultimately evaluating weld quality and joint performance.

#### 3.1.4. Influence of the Temperature Field on the Formation of the Effective Fusion Zone

During electrofusion welding, the evolution of the temperature field directly governs the formation and expansion of the effective molten zone (fusion zone), which in turn critically influences the mechanical performance and microstructural characteristics of the welded joint. The formation of an effective molten zone not only requires the temperature to exceed the material’s melting point, but also for it to be maintained for a sufficient duration to allow adequate diffusion and interpenetration of polymer chains, thereby establishing a continuous interface with mechanical properties comparable to those of the base material [10,31]. Ammosova [32] demonstrated that the spatial distribution, gradient magnitude, and temporal evolution of the temperature field are all key factors governing molten zone formation.

**Figure 8:**
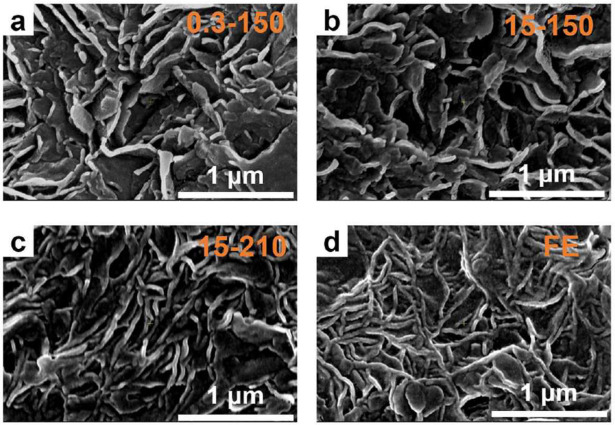
Scanning electron microscopy (SEM) images of amorphous-phase etched thin sections for samples with different entanglement densities [34]. (**a**) Low entanglement density sample (0.3–150), (**b**) Medium entanglement density sample (15–150), (**c**) Medium-high entanglement, (**d**) Fully entangled sample (FE).

Building upon the established role of the temperature field, subsequent studies have further explored, from a quantitative perspective, how its spatial distribution controls the boundary and morphology of the molten zone. Najafabadi [33] reported, through both numerical simulations and experimental investigations, that the spatial distribution of the temperature field directly determines the extent of the molten zone. Finite element heat transfer analyses indicate that only when the temperature at the fitting–pipe interface reaches and sustains the melting point for an adequate duration can the molten zone fully bridge the interface, forming a complete fusion structure. Otherwise, localized regions of insufficient melting—commonly referred to as “cold welds”—may occur. These findings highlight the critical role of both the peak temperature location and the temperature gradient in governing the formation of an effective molten zone. As shown in Figure 8, variations in entanglement density lead to distinct microstructural features within the fusion zone, as pointed out by Wang [34].

Obedan [21] systematically investigated the heat transfer behavior during electrofusion welding and its underlying influence on molten zone formation. The study demonstrates that the evolution of the temperature field governs the initiation location, propagation direction, and final thickness of the molten zone. Only when the local temperature exceeds the melting point of polyethylene and is maintained for a sufficient duration can complete melting occur at the interface, enabling the formation of a continuous bonded region.

Furthermore, this study emphasizes that limitations in heat transfer or non-uniform temperature distribution can result in discontinuities or insufficient thickness of the molten zone, thereby adversely affecting the structural integrity and mechanical performance of the welded joint. By elucidating the fundamental role of heat transfer and temperature field evolution in molten zone formation, this work provides important theoretical support for subsequent optimization of welding parameters and precise control of the thermal field during electrofusion welding.

Although numerous studies have demonstrated that numerical simulation plays an important role in predicting temperature distribution and fusion zone evolution during electrofusion welding, noticeable discrepancies still exist among different research results due to variations in material parameters, boundary condition assumptions, and heat source modeling approaches [24]. Some studies mainly focus on macroscopic thermal behavior [35], whereas others place greater emphasis on local interfacial phenomena and thermo-mechanical coupling effects [36]. Compared with purely experimental approaches [18], numerical simulation exhibits stronger advantages in process optimization and parameter sensitivity analysis [37]; however, its prediction accuracy still highly depends on the constitutive material models employed and the quality of experimental validation [38]. Therefore, future studies should further strengthen the integration of multi-physics simulations, in situ monitoring, and experimental characterization in order to improve model reliability and engineering applicability.

#### 3.1.5. Practical Recommendations for Reducing Temperature Field Inhomogeneity

The aforementioned studies indicate that temperature field non-uniformity is one of the principal factors leading to abnormal fusion zone formation, residual stress concentration, and the development of typical defects during electrofusion welding. When the circumferential or radial temperature distribution within the joint becomes unbalanced, localized regions may undergo overheating and thermal degradation, whereas regions farther away from the heat source may experience insufficient heating, resulting in incomplete fusion or cold welding defects. These phenomena ultimately reduce the mechanical performance and long-term service reliability of welded joints. Therefore, in engineering practice, reducing temperature field non-uniformity through process optimization and structural modification has become a key strategy for improving the quality of electrofusion joints.

First, the structural parameters and arrangement of the heating wire should be optimized. Rajabi [39] investigated electrofusion welding optimization through conjugate heat transfer simulations combined with the Taguchi method and demonstrated that variations in heating wire spacing, embedding height, and wire diameter significantly affect the internal heat transfer capability and temperature distribution uniformity within the joint. Specifically, excessively small wire spacing tends to cause localized heat accumulation and excessive melt flow, whereas excessively large spacing may generate cold zones that are unfavorable for sufficient interfacial fusion. Appropriately increasing the position of the heating wire and optimizing the wire diameter can improve the heat transfer efficiency toward the welding interface, thereby promoting a more uniform temperature field distribution. Therefore, during the design stage of electrofusion fittings, arrangement density, embedding depth, and winding pitch of resistance wires should be reasonably determined according to pipe diameter and wall thickness in order to avoid localized heat concentration and thermal blind zones.

Secondly, welding input parameters should be properly controlled. Doaei [40] reported that welding voltage, current, and heating duration directly determine the heat input level per unit time and are among the most sensitive process variables affecting the temperature field. When the heat input is insufficient, the thickness of the molten layer becomes limited and interfacial molecular diffusion remains inadequate, thereby increasing the likelihood of cold welding defects. Conversely, excessive heat input may result in thermo-oxidative degradation of polyethylene, melt overflow, and structural deformation. In engineering practice, welding parameters should be selected according to material grade, environmental temperature, wall thickness, and fitting specifications, rather than applying a universal parameter set to all conditions. For large-diameter or thick-walled joints, strategies such as staged heating, pulse heating, or adaptive compensation can be employed to reduce radial temperature differences and improve overall fusion consistency.

Thirdly, assembly accuracy and interfacial contact quality should be improved. Even when heat input conditions are appropriate, insufficient pipe insertion, eccentric assembly, excessive interfacial gaps, or surface contamination may disrupt the heat conduction pathway at the interface, leading to abnormal local temperature rise and the formation of non-uniform fusion zones. Nie [36] demonstrated that a distinct structural transition region exists between the welding zone and the base material, and its formation is jointly influenced by thermal history and interfacial flow behavior, indicating that interfacial contact conditions play a crucial role in fusion zone evolution. Therefore, strict procedures—including oxide layer scraping, alcohol cleaning, coaxial alignment, and standardized insertion depth control—should be implemented prior to welding in order to ensure stable and uniform heat transfer conditions.

Fourthly, real-time monitoring and intelligent feedback technologies should be introduced. With the development of infrared thermography, embedded temperature sensors, and digital controllers, online monitoring of surface temperature fields and heating conditions during welding has become feasible. Shen [24] demonstrated that by identifying abnormal temperature rise regions in real time and dynamically adjusting power output, conventional open-loop welding can be upgraded into a closed-loop thermal management process, thereby significantly reducing quality fluctuations caused by temperature field non-uniformity.

Overall, reducing temperature field non-uniformity requires coordinated implementation from four aspects: structural design, parameter matching, assembly quality, and intelligent monitoring. Future development trends in electrofusion welding technology are expected to gradually shift from experience-based parameter control toward digitalized, refined, and adaptive thermal field regulation, thereby providing more reliable technical support for high-integrity polymeric pipeline connections.

### 3.2. Characteristics of the Fusion Zone and Its Influence on Welding Defects

Under the influence of a non-uniform temperature field, the material within the welding region does not enter the molten state simultaneously or uniformly; instead, a molten zone with distinct spatial extent and heterogeneous thermal states is formed. The thickness, continuity, and thermal history of this molten zone directly determine the quality of interfacial bonding and play a critical role in the formation of welding defects [41].

#### 3.2.1. Thickness and Melting State Characteristics of the Fusion Zone

During electrofusion welding, the effective molten zone (melted fusion zone) is not only the critical region for joint formation, but its thickness and melting state are directly related to weld continuity, microstructural evolution, and mechanical performance. The formation of the molten zone is governed by both the spatial distribution and temporal evolution of the temperature field. When the temperature at the interface exceeds the melting point of the material and is maintained for a sufficient duration, polymer chains at the interface undergo diffusion and interpenetration, leading to a progressive increase in the molten zone thickness and ultimately the formation of a continuous welded structure. Awadi [42] demonstrated that both the level of heat input and heat transfer behavior significantly influence the thickness of the molten zone. In general, higher welding temperatures and longer heat transfer durations tend to increase the molten zone thickness; however, this relationship is not linear, as it is jointly affected by material thermophysical properties, geometric factors, and external processing conditions. Furthermore, experimental results reported by Zhang [43] indicated that the molten zone is not a homogeneous melt. Instead, it exhibits microstructural heterogeneity due to variations in temperature distribution and cooling conditions, leading to distinct microstructural features in different regions of the weld.

In thick-walled fittings, heat diffusion toward the outer layers is significantly constrained, resulting in the molten zone being concentrated primarily near the interface, while the base material farther from the heat source exhibits a comparatively smaller molten thickness. Zeng [44] reported that adjustments in welding parameters—such as heat input intensity and heating duration—can either mitigate or exacerbate this non-uniform thickness distribution. This finding further underscores that the control of molten zone thickness by the temperature field exhibits a complex spatiotemporal coupling behavior.

In summary, the thickness and state of the molten zone are governed by the combined effects of the spatial distribution of the temperature field, the thermal response of the material, and the dynamic evolution of welding parameters (as shown in Table 1). Quantitative analysis and mechanistic understanding of these factors not only facilitate the prediction of weld geometry and microstructural evolution, but also provide a scientific basis for process optimization aimed at achieving high-quality welded joints.

#### 3.2.2. Typical Welding Defects Induced by Abnormal Fusion Zone Behavior

When the formation of the molten zone deviates from the ideal state due to variations in the temperature field or processing conditions, welding defects are likely to arise. The integrity and state of the molten zone are central to the quality of electrofusion welded joints. Any abnormalities in this region can readily lead to the formation of various typical defects within the weld, which not only degrade the mechanical performance of the joint but may also result in leakage or even catastrophic failure during service. Wang [47] demonstrated that defects in electrofusion welding can be classified across multiple scales, from microstructural to macroscopic levels, and are primarily induced by factors such as incomplete fusion, insufficient heating, excessive melt flow, or structural deformation. Typical defects associated with the molten zone are summarized in Table 2.

#### 3.2.3. Reasonable Ranges of Process Parameters Based on Literature

Existing studies generally agree that the quality of electrofusion welded joints is not determined by a single process parameter, but rather by the combined effects of voltage (or current), heating time, cooling conditions, and their mutual compatibility. A substantial body of experimental and engineering literature emphasizes that, instead of defining a universal “optimal parameter value,” welding parameters should be maintained within a suitable process window that accounts for variations in pipe diameter, material grade, and environmental conditions, thereby ensuring adequate formation of the molten zone while minimizing the risk of defects [50].

From the perspective of heat input, Shi [51] pointed out that electrofusion welding is fundamentally a controlled heat transfer process in which the appropriate parameter range should ensure that the interface region reliably reaches and maintains the material’s melting temperature. Subsequent studies have further highlighted the coupled relationships among different process parameters. For example, Shi [52] demonstrated that heating time and voltage are not independent variables. Under a given voltage, increasing the heating time can enlarge the thickness of the molten zone; however, at higher voltage levels, the same heating duration may significantly increase the risk of overheating. This indicates that the concept of a suitable process window should be understood in terms of parameter combinations rather than individual parameter thresholds.

**Figure 9:**
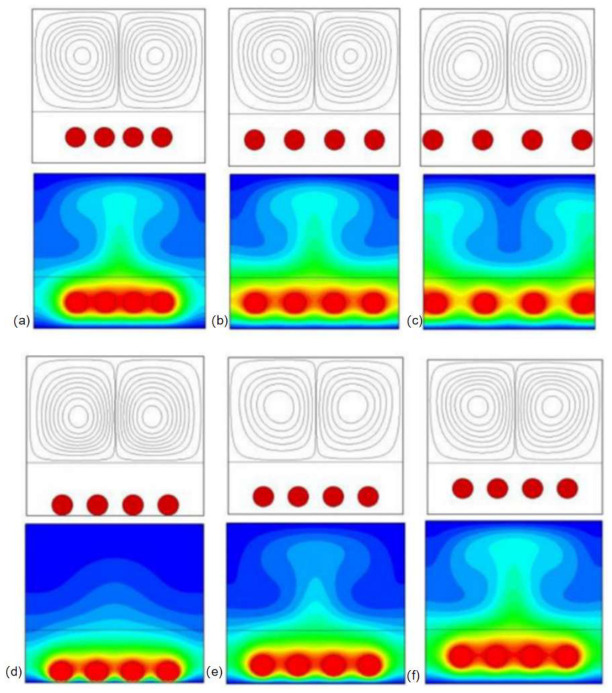
Streamlines and isotherm distributions under different heating wire spacings and embedding heights [39]. (**a**) d = 25, Nu_m_ = 2.31, (**b**) d = 35, Nu_m_ = 2.52, and (**c**) d = 43, Nu_m_ = 2.52 (**d**) h = 10, Nu_m_ = 0.831, (**e**) h = 17, Nu_m_ = 1.689, and (**f**) h = 25, Nu_m_ = 2.502.

A synthesis of the existing literature indicates that the appropriate range of electrofusion welding parameters should not be regarded as a fixed set of values, but rather as a multidimensional process window jointly constrained by material properties, geometric conditions, environmental factors [4], and quality evaluation criteria. The process-oriented workflow for real-time quality monitoring during electrofusion welding is illustrated in Figure 9. Systematic characterization and consolidation of these parameter ranges not only help to reconcile discrepancies among different studies, but also provide a critical foundation for subsequent process optimization strategies based on temperature field evolution, molten zone development, and defect control.

#### 3.2.4. Modern Methods for Defect Diagnosis and Quality Evaluation of Electrofusion Joints

The aforementioned studies indicate that abnormal fusion zone behavior is one of the principal causes of typical defects in electrofusion joints, including cold welding, voids, over-welding, and structural deformation. Once such defects are formed, they not only weaken the load-bearing capacity and sealing performance of the joint, but may also induce crack propagation, medium leakage, and failure accidents during long-term service. Therefore, merely identifying defect formation mechanisms is insufficient to satisfy engineering application requirements. Rapid and accurate defect detection, together with scientific evaluation of joint quality, has become an important research direction in the field of electrofusion welding. Different inspection technologies exhibit distinct characteristics in terms of operating principles, applicability, and engineering cost. Their major advantages and limitations are summarized in Table 3.

Overall, different inspection technologies exhibit distinct strengths and demonstrate clear complementarity. Visual inspection is suitable for rapid preliminary screening, conventional ultrasonic testing is appropriate for on-site internal defect identification, and phased array ultrasonic testing is more suitable for high-precision quality evaluation. Infrared thermography is advantageous for real-time monitoring during the welding process, whereas radiographic testing and industrial CT are more appropriate for high-precision internal structural analysis. Destructive mechanical testing remains an important method for final performance verification. Future development trends in electrofusion joint quality control are expected to move toward a comprehensive evaluation framework integrating “online monitoring + non-destructive testing + artificial intelligence diagnosis + sampling-based mechanical verification,” thereby achieving full-process and highly reliable quality assurance.

Existing studies on defect diagnosis and quality evaluation still exhibit significant differences in terms of detection principles, sensitivity, and applicability. Traditional non-destructive testing methods, such as ultrasonic testing [53] and infrared inspection [24], generally demonstrate relatively stable defect identification capability; however, they still show certain limitations in recognizing early-stage interfacial defects and complex multi-defect coupling phenomena [9]. In contrast, recently developed artificial intelligence-based diagnostic approaches have demonstrated greater potential in pattern recognition and real-time analysis [54]. Nevertheless, their reliability is still constrained by issues including limited dataset size, insufficient model generalization capability, and the lack of unified evaluation standards [55]. Therefore, future research should not only further improve diagnostic accuracy, but also focus on establishing unified defect databases, interpretable learning frameworks, and multi-source data fusion strategies, in order to enhance the robustness and engineering applicability of intelligent diagnostic systems.

### 3.3. Thermo-Mechanical Coupling Effects and Residual Stress in Joints

Electrofusion welding is not only a thermal process but also a thermo-mechanically coupled process accompanied by significant structural responses. During the cooling and solidification of the molten material, the combined effects of temperature gradients, variations in material properties, and structural constraints inevitably lead to the development of residual stresses within the welded joint [19]. Although these stresses are not directly observable after welding, they can exert a substantial influence on the mechanical performance and long-term service behavior of the joint.

#### 3.3.1. Cooling Shrinkage and Structural Constraint Conditions

During electrofusion welding and other thermofusion joining processes, welded joints undergo heating, melting, and subsequent cooling stages. Owing to the non-uniform temperature field and the inherent shrinkage behavior of materials, significant cooling-induced shrinkage strain is generated, leading to the formation of residual stresses in the weld zone and its surrounding regions. As reported by Poduška [56], such residual stresses are not induced by external loads; rather, they originate within the weld and heat-affected zone as a result of the combined effects of non-uniform cooling shrinkage, structural constraints, and thermo-elasto-plastic material responses. The magnitude and distribution of these stresses directly influence the mechanical performance, fatigue resistance, and long-term stability of the welded joints.

**Figure 10:**
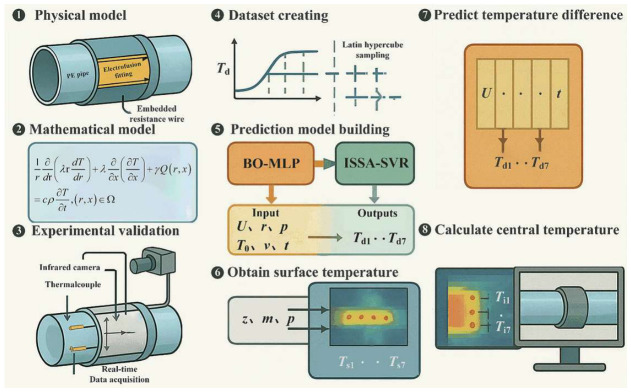
The quality monitoring process for welding of PE electrofusion fittings [27].

For semi-crystalline thermoplastics such as polyethylene, pronounced volumetric changes occur during the cooling process. The coefficient of thermal expansion differs significantly between the molten and solidified states, resulting in inconsistent thermal contraction rates across different regions. This mismatch in shrinkage behavior gives rise to strain incompatibility within the material. Gao [57] further demonstrated that, as the material cools from elevated temperatures to the solid state, local temperature gradients and non-uniform shrinkage are among the primary driving factors for residual stress development. This phenomenon is intrinsically associated with the thermo-mechanical coupling behavior within the temperature field. As illustrated in Figure 10, the thermo-mechanical coupling behavior and residual stress distribution in the joint.

Building upon this understanding, existing studies further indicate that residual stresses induced by cooling shrinkage cannot be attributed to a single thermal effect; rather, they represent the combined outcome of multiple material and structural factors. Deveci [58] emphasized that residual stresses arising from cooling shrinkage and structural constraints are influenced not only by the evolution of the temperature field during welding, but also by the thermo-elasto-plastic properties of the material, welding parameters (such as heating temperature and heating time), and external constraint conditions (e.g., fixture configuration and cooling media) [59]. Therefore, a comprehensive understanding of the thermo-mechanical coupling effects associated with cooling shrinkage is essential for accurately predicting the residual stress distribution in electrofusion-welded joints, as well as for evaluating weld performance and long-term reliability. This aspect also constitutes a key focus in both numerical simulation and experimental investigations of polymer welding processes.

#### 3.3.2. Formation Mechanisms of Residual Stress Under Thermo-Mechanical Coupling

The combined effects of temperature gradients, cooling rates, and structural constraints give rise to a complex thermo-mechanical coupling response within electrofusion-welded joints. Consequently, the formation of residual stresses during welding represents a typical thermo-mechanical coupling process. In electrofusion welding and other thermoplastic fusion joining techniques, the weld region undergoes intense heating, melting, and subsequent rapid cooling. During this process, the non-uniform temperature field, mismatched thermal expansion and contraction, and the thermo-elasto-plastic response of the material collectively lead to the generation of residual stresses that cannot be fully eliminated. In simple terms, materials expand upon heating and contract during cooling; however, due to differences in contact conditions and constraints across regions, thermal strains cannot be fully accommodated, resulting in the accumulation of internal stresses and ultimately the formation of residual stresses.

This thermo-mechanical coupling mechanism has progressed beyond qualitative understanding, with extensive experimental observations and numerical analyses in recent years systematically validating the key driving factors governing residual stress formation. Falodun [60] demonstrated through both review and combined experimental–numerical studies that temperature gradients and inconsistencies in thermal expansion are the primary driving forces behind residual stress generation. When the welding heat source elevates the temperature of the weld zone while surrounding regions remain relatively cooler, the weld material expands first. During the subsequent rapid cooling stage, the outer layers tend to contract more quickly than the inner regions, leading to asynchronous cooling and strain incompatibility—where different parts of the material cannot freely expand or contract. As a result, irreversible internal stresses are generated. This mismatch in thermal expansion, along with its nonlinear evolution with temperature, constitutes the fundamental mechanism of residual stress formation under thermo-mechanical coupling conditions.

**Figure 11:**
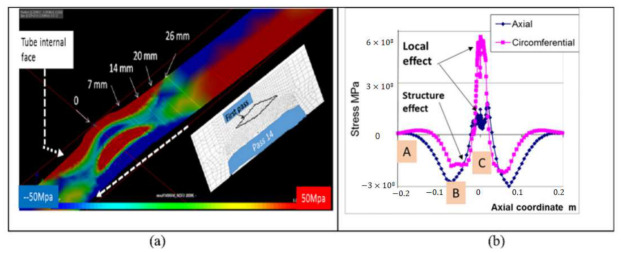
(**a**) Finite element mesh for 14 passes of welding for a tube with a chamfer. (**b**) Simulated axial (longitudinal) and circumferential weld residual stress versus axial coordinate for a tube with a chamfer with 13 passes [64].

In addition, the formation of residual stresses is strongly associated with history dependence, meaning that the thermal cycles and loading paths experienced by the material during the welding process influence the evolution and accumulation of stresses. Regarding typical residual stress evolution and its influence on joint integrity, as investigated in the works (Figure 11) of S. Taheri [61], J.A. Le Duff [62], and Zaza [63], it was demonstrated that even in the absence of plastic deformation, materials can retain internal stress “memory” due to mismatches in thermal expansion and differences in loading history. This arises from strain incompatibility, whereby different regions of the material undergo unequal thermal strains during heating and cooling. As a result, residual stresses can persist even without permanent deformation. Such a mechanism is widely observed not only in welding processes but also in other thermally driven manufacturing techniques, including additive manufacturing.

#### 3.3.3. Potential Effects of Residual Stress on Joint Performance and Service Behavior

Although residual stresses are difficult to observe directly after welding, they can have a significant impact on the mechanical performance and long-term service behavior of welded joints. Numerous experimental and numerical studies on thermoplastic pipeline materials, particularly polyethylene, have demonstrated that residual stresses influence both the static strength and long-term stability of joints. Tan [65] employed the ring-cutting method to measure the residual stress distribution in HDPE pipes and found that different polyethylene pipes exhibited markedly different residual stress levels under identical conditions. These differences led to variations in yield behavior and strength during quasi-static tensile tests [66]. When residual stresses are tensile in nature, they can promote earlier attainment of failure conditions under external loading, thereby increasing the likelihood of premature fracture or plastic deformation [36,67].

**Figure 12:**
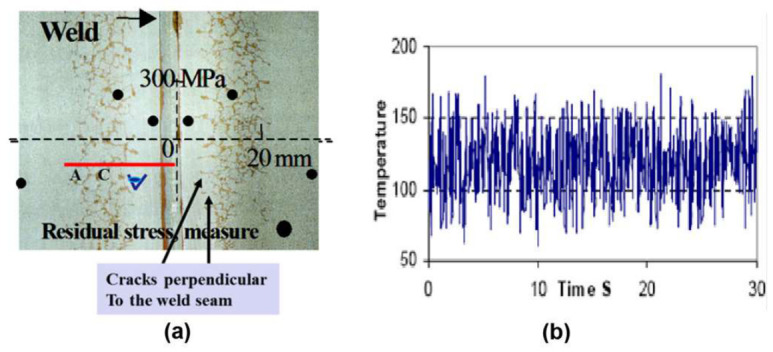
(**a**) Crack induced by welding residual stress along the longitudinal seam; (**b**) partial temperature fluctuations in the heat dissipation system model [64].

**Figure 13:**
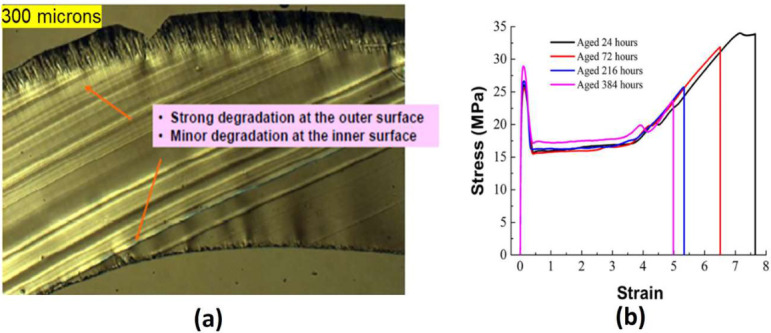
(**a**) Surface embrittlement of aged polyethylene pipes; (**b**) stress–strain curves of polyethylene pipes after thermal aging at 95 °C under cyclic pressure loading [1].

During long-term service, residual stresses may superimpose with external loads, accelerating creep damage and progressive material degradation [68]. In particular, tensile residual stresses can elevate the effective local stress when combined with operational loads, which is critical in fatigue and crack propagation analyses [69]. Leggatt [70] reported that even under relatively low external loads, residual stresses can shift the local stress state toward more unfavorable conditions, significantly lowering the threshold for crack initiation, reducing fatigue life, and ultimately compromising structural reliability [71]. Figure 12 presents the long-term performance and reliability assessment framework of welded joints [1]. Furthermore, long-term thermal aging and cyclic pressure loading can significantly accelerate the deterioration of polyethylene materials. As shown in Figure 13, surface embrittlement becomes increasingly evident after aging, accompanied by a gradual degradation of the stress–strain response, indicating a reduction in ductility and structural reliability during long-term service.

In summary, the potential effects of residual stresses on welded joints can be primarily reflected in the following aspects:

(1) Static mechanical performance: Tensile residual stresses can reduce the tensile strength and yield strength of the material, making the joint more susceptible to premature failure under external loading [72].

(2) Fatigue performance: Tensile residual stresses accelerate crack initiation and propagation, significantly shortening the fatigue life of the joint [73].

(3) Fracture toughness and crack propagation: In the presence of initial defects, residual stresses increase the stress intensity factor, thereby reducing fracture ductility and promoting unstable crack growth.

(4) Environmental sensitivity during service: Tensile residual stresses may amplify susceptibility to stress corrosion, making joints more prone to failure under the combined action of corrosive environments and mechanical loading [74].

Therefore, understanding and controlling residual stresses is of great importance for extending the service life of welded joints, improving structural reliability, and guiding the optimization of manufacturing processes. As a result, residual stress has become an increasingly important focus in both research and engineering practice of electrofusion welding and other thermoplastic welding technologies.

#### 3.3.4. Practical Recommendations for Reducing Residual Stresses in Welded Joints

In summary, residual stresses primarily originate from non-uniform heating, differential cooling shrinkage, and structural constraints within the joint region during the welding process. Excessive residual stresses can readily lead to stress concentration, interfacial cracking, and long-term service failure. Therefore, adopting effective measures to reduce residual stresses is of great importance for improving the reliability of electrofusion joints.

**Figure 14:**
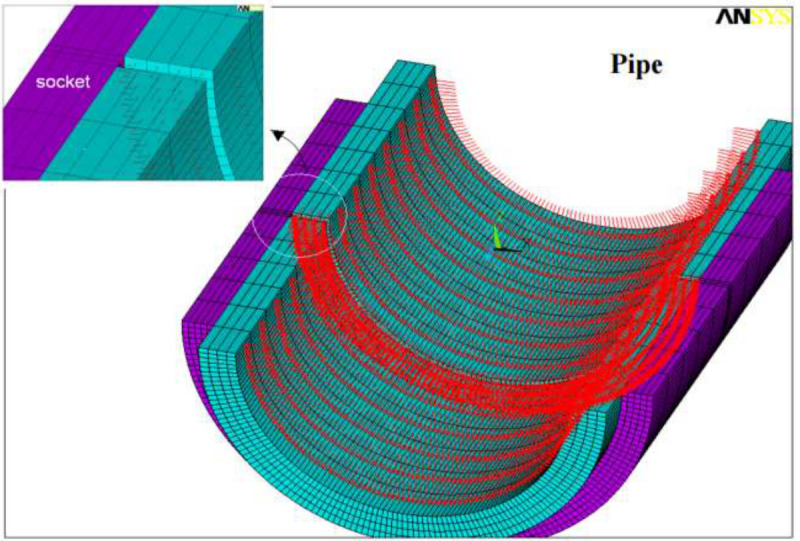
Typical finite element model of pipe and socket subjected to internal pressure [37].

First, heat input and cooling rate should be properly controlled. The larger the temperature gradient, the more pronounced the shrinkage stress after cooling. Khademi-Zahedi et al. [37] demonstrated that temperature variations significantly influence the axial stress and equivalent stress of electrofusion socket joints, with higher stress levels observed under low-temperature conditions. Therefore, matched welding voltage, current, and heating duration should be strictly adopted to avoid overheating or insufficient heating. For large-diameter pipelines, staged heating and natural slow cooling strategies may be employed to reduce thermal stress concentration. Finite element analysis further illustrates the stress distribution characteristics within electrofusion joints under internal pressure loading, as shown in Figure 14. The results indicate that stress concentration is more likely to occur near the socket–pipe interface, highlighting the importance of optimizing thermal management and structural design to mitigate residual stress accumulation.

Secondly, joint structure and material compatibility should be optimized. DebRoy et al. [75] reported that sleeve ends, wall-thickness transition regions, and stiffness variation zones are typically locations of residual stress concentration. Finite element analysis further indicated that local geometric variations can increase stress levels within the joint region. Therefore, smooth transition structures and appropriate wall-thickness designs should be adopted, while ensuring that the sleeve and base material possess similar coefficients of thermal expansion and comparable mechanical properties.

Thirdly, assembly quality should be improved. Misalignment, eccentric assembly, forced fitting, and insufficient insertion depth may introduce additional stresses after welding. During construction, pipe sections should be maintained in coaxial alignment with consistent insertion depth, and forced correction using external loads should be avoided.

Fourthly, post-weld cooling management should be emphasized. Premature movement, backfilling, or pressurization before sufficient cooling of the joint can easily result in stress superposition and structural damage. Therefore, subsequent operations should only be conducted after completion of the prescribed cooling period according to relevant standards. Under cold environmental conditions, thermal insulation and controlled slow-cooling measures may also be adopted.

Fifthly, the influence of external service loads should be minimized. For buried pipelines, the stress level within the joint region is usually higher than that in straight pipe sections due to the combined effects of soil pressure, vehicle loads, internal pressure, and thermal cycling. Therefore, backfilling quality, burial conditions, and operating pressure fluctuations should be optimized to reduce the coupling effect between external loads and residual stresses.

Overall, reducing residual stresses should primarily focus on five aspects: process parameter control, structural optimization, assembly precision, cooling management, and external load regulation, thereby improving the long-term service safety of electrofusion joints.

## 4. Development Trends in Electrofusion Welding of Polymeric Pipelines

Integrating the results of bibliometric analysis with recent advances in technical research, it can be observed that electrofusion welding of polymeric pipelines is transitioning from an engineering stage dominated by empirical parameter control toward a more refined development stage centered on mechanism-based understanding and process controllability [76]. The evolution of research trends in this field is summarized in Table 4.

The future development of electrofusion welding for polymeric pipelines is expected to focus on the following key aspects:

**(1) Transition from Empirical Approaches to Mechanism-Driven and Process-Coordinated Welding Design**

Future research in electrofusion welding will gradually shift from empirical parameter-based optimization toward mechanism-driven, quantitative, and predictive process design frameworks [78]. On the one hand, increasing attention will be given to fundamental material–process interactions, including thermophysical properties of materials [34], crystallization kinetics [79], and interfacial molecular diffusion mechanisms. These factors will be systematically incorporated to describe heat input, melt evolution, and interfacial bonding behavior during welding, thereby establishing a more physically meaningful relationship between welding parameters and joint performance. This trend indicates that traditional empirical optimization relying on single-parameter windows will be progressively replaced by multi-factor coordinated process design strategies [80].

On the other hand, with the deepening understanding of welding mechanisms, precise control of the temperature field and quantitative characterization of the effective fusion zone will become essential components of mechanism-driven process design. By optimizing the structure of heating elements, heating paths, and feedback control strategies, it will be possible to improve the stability and repeatability of temperature distribution in the welding region. Combined with thermal analysis and structural characterization techniques, the thickness, morphology, and spatial uniformity of the fusion zone can be quantitatively evaluated, providing more reliable physical criteria for weld quality assessment.

Meanwhile, research perspectives will further extend to structural responses under thermo-mechanical coupling. By analyzing the formation and evolution of cooling-induced shrinkage and residual stresses, and linking these factors to long-term failure modes such as creep behavior, slow crack growth, and environmental stress cracking [81], electrofusion welding research is expected to shift from “joint strength evaluation” toward “full-life reliability assessment.”

In this context, the focus of defect research is also transitioning from post-characterization to process-oriented prevention. Through dynamic regulation of heat input based on temperature field evolution, standardized control of interfacial conditions, and coordinated optimization of process parameters, it becomes possible to constrain defect initiation conditions in advance, thereby reducing the probability of welding defects at a system level [82]. This transition signifies that electrofusion welding is evolving from experience-based “passive correction” to mechanism-informed “active control” [83,84].

Electrofusion welding technology is currently undergoing a critical transition from traditional experience-based process optimization toward mechanism-driven intelligent manufacturing. Although significant progress has been achieved in recent years in areas such as welding parameter control, numerical simulation, and defect diagnosis, existing studies still exhibit strong fragmentation characteristics, and most investigations remain primarily at the laboratory-scale research stage. The lack of a unified multi-physics analytical framework and long-term service validation remains one of the major limiting factors preventing many current research achievements from being effectively translated into practical engineering applications.

**(2) Development of Intelligent Defect Recognition Based on Deep Learning and Advanced Inspection Technologies**

With the large-scale application of polymeric pipeline systems and increasing requirements for quality consistency, intelligent defect recognition technologies based on advanced algorithms are emerging as a key development direction in electrofusion welding. In recent years, the integration of deep learning methods with non-destructive testing (NDT) techniques has provided new approaches for weld quality evaluation. For example, automated classification models based on phased-array ultrasonic data, as well as intelligent defect screening methods combining thermal imaging information with clustering algorithms [27], have demonstrated high recognition accuracy across different weld types and scales. These studies highlight the advantages of deep learning in processing high-dimensional and multi-modal inspection data.

Furthermore, defect recognition techniques based on deep neural networks [55], attention mechanisms, and sequential modeling methods are capable not only of efficient post-weld defect identification but also of extending toward real-time quality monitoring and intelligent decision support during the welding process [85]. In the future, with the continuous accumulation of inspection data and further improvement in algorithmic models, intelligent inspection technologies are expected to be deeply integrated with welding equipment control systems. This integration will provide critical support for intelligent manufacturing and full-process quality control in electrofusion welding of polymeric pipelines.

Future breakthroughs in the field of electrofusion welding will largely depend on the deep integration of thermo-mechanical coupling analysis, real-time sensing technologies, data-driven modeling, and intelligent process control. In particular, the combination of artificial intelligence with multi-source monitoring data is expected to fundamentally transform traditional welding quality evaluation methods, enabling the transition from conventional “post-weld defect inspection” toward predictive and adaptive process regulation.

Furthermore, with the rapid development of hydrogen transportation infrastructure and intelligent pipeline network systems, the long-term reliability of polymeric welded joints under complex service environments is expected to become one of the most important research directions in the future. Compared with conventional short-term mechanical performance evaluation, future studies should place greater emphasis on issues such as life prediction, environmental aging behavior, creep–fatigue coupling, and structural integrity assessment under multi-factor coupling conditions.

Overall, future development of electrofusion welding technology should not only focus on improving welding quality itself, but also establish a full life-cycle intelligent manufacturing and reliability evaluation framework encompassing material design, process control, defect diagnosis, and long-term service performance assessment.

## 5. Practical Challenges and Limitations of Electrofusion Welding

Although significant progress has been achieved in recent years in the field of electrofusion welding for polymeric pipelines, several practical limitations and engineering challenges still remain in large-scale industrial applications. First, welding quality is highly sensitive to external conditions, including pipe surface contamination [48], assembly misalignment, environmental temperature fluctuations [56], and variations in operator workmanship. These factors can significantly influence heat distribution uniformity and fusion zone stability, thereby increasing the risk of defects such as cold welding, porosity, and incomplete fusion.

Secondly, although numerical simulation and intelligent monitoring technologies have developed rapidly in recent years, most current approaches still remain primarily at the laboratory research stage. Their reliability, robustness, and real-time adaptability under complex field conditions have not yet been sufficiently validated [86]. In particular, the lack of large-scale defect datasets and unified evaluation standards has limited the industrial implementation of artificial intelligence-based intelligent diagnostic systems.

Thirdly, electrofusion welded joints still face challenges related to residual stress accumulation, long-term creep behavior, and environmental aging effects. Under severe service conditions such as hydrogen transportation, cyclic loading, and temperature fluctuations, these issues may directly affect the long-term structural integrity and service reliability of welded joints [87].

In addition, factors such as relatively high equipment cost, strict installation requirements, and insufficient standardization among different material systems and pipeline configurations also limit the widespread adoption of electrofusion welding technology to a certain extent. Therefore, future research should further strengthen the integration of mechanism-driven process control, intelligent quality monitoring, standardized evaluation systems, and long-term reliability assessment in order to improve the engineering applicability and industrial implementation capability of electrofusion welding technology.

From the perspective of industrial application, the scalability, economic feasibility, and sustainability of electrofusion welding technology are also critical issues that require further attention in future development. Although electrofusion welding exhibits significant advantages in sealing performance, construction flexibility, and joint reliability, its relatively high equipment cost, strict operational requirements, and strong dependence on operator experience may increase the overall implementation cost in large-scale infrastructure projects. Furthermore, differences in material compatibility, insufficient standardization of fittings, and complex field construction conditions further increase the difficulty of achieving large-scale industrial deployment across diverse pipeline systems.

Nevertheless, with the continuous global growth in demand for low-carbon energy transportation and intelligent infrastructure systems, electrofusion welding technology still demonstrates considerable long-term sustainable development potential. Compared with traditional metallic pipeline systems, polymeric pipelines generally offer advantages including corrosion resistance, light weight, low maintenance requirements, and lower life-cycle energy consumption. Moreover, the further integration of intelligent monitoring technologies, automated process control, and data-driven quality evaluation methods is expected to improve production efficiency, reduce operational costs, and further enhance the long-term sustainability of electrofusion welding technology in future industrial applications.

## 6. Conclusions

This review systematically summarized the recent advances in electrofusion welding of polymeric pipelines by integrating bibliometric analysis with mechanism-oriented technical analysis. The main conclusions are summarized as follows:

(1) Based on bibliometric analysis of publications from 2015 to 2025, the research field of electrofusion welding has exhibited continuous growth and strong interdisciplinary characteristics. Current research hotspots are mainly concentrated on HDPE material adaptability, welding parameter optimization, temperature field evolution, defect formation mechanisms, residual stress behavior, and long-term reliability evaluation.

(2) Existing studies demonstrate that the temperature field evolution plays a dominant role in molten zone formation and joint quality. The spatial non-uniformity of temperature distribution directly affects polymer chain diffusion, interfacial bonding behavior, and molten zone continuity, thereby significantly influencing the mechanical performance and structural integrity of welded joints.

(3) The formation of welding defects is closely associated with abnormal molten zone evolution and improper process conditions. Typical defects such as cold welding, voids, overheating, and structural deformation are primarily induced by insufficient heat input, excessive local heating, asymmetric thermal diffusion, and poor assembly conditions.

(4) Thermo-mechanical coupling and cooling-induced shrinkage are identified as the major mechanisms responsible for residual stress generation in electrofusion joints. Residual stresses can significantly affect crack initiation, fatigue behavior, creep resistance, and long-term service reliability, especially under complex operating conditions such as hydrogen transportation and cyclic loading environments.

(5) Compared with traditional empirical welding control approaches, current research is progressively transitioning toward mechanism-driven process regulation based on numerical simulation, multi-physics coupling analysis, intelligent sensing, and real-time monitoring technologies. Emerging intelligent diagnostic methods based on machine learning and multi-source data fusion also demonstrate strong potential for future industrial applications.

(6) Despite significant progress in recent years, electrofusion welding still faces several practical challenges, including sensitivity to environmental conditions, insufficient standardization, limited robustness of intelligent diagnostic systems, and uncertainties associated with long-term service behavior under harsh environments.

(7) Future research should further focus on thermo-mechanical multi-field coupling mechanisms, intelligent process control, digital and adaptive welding technologies, AI-assisted defect diagnosis, and full-lifecycle reliability assessment. In particular, with the rapid development of hydrogen transportation infrastructure and intelligent pipeline systems, improving the long-term structural integrity and intelligent quality assurance capability of electrofusion welded joints will become one of the most important research directions in the future.

## Figures and Tables

**Table 1:** Influence of Fusion Zone Thickness on Welding Quality.

Impact Category	Description of Effects	Mechanism/Cause	Representative Studies/Cases
Local Mechanical Properties	Significant reduction in the static mechanical performance of the joint, including tensile strength and peel strength; maximum load capacity and elongation at break decrease markedly	Defects act as sources of stress concentration, accelerating crack initiation and propagation, leading to intensified local stress concentration and enlarged plastic deformation zones	M. Kiani et al. conducted tensile and peel tests on polyethylene (PE) electrofusion joints; studies on pipeline joint performance prediction based on numerical simulation and experiments [7]
Fatigue Life	Fatigue life is reduced by more than an order of magnitude compared with defect-free joints; premature fatigue failure occurs	Stress concentration at defect locations facilitates crack initiation under cyclic loading and accelerates crack propagation; sharp defect edges significantly increase stress intensity factors	Fatigue testing of HDPE electrofusion joints; studies combining finite element analysis with fatigue failure criteria [45]
Long-Term Service Behavior	Degradation of long-term reliability; increased susceptibility to environmental stress cracking (ESC) and performance deterioration; accelerated local degradation and material embrittlement	Microvoids and incomplete fusion interfaces in defect regions become preferential pathways for media permeation; welding defects act as sites for corrosive media accumulation	Studies on long-term hydrothermal aging of polyethylene joints; research on fluid transport pipelines [46]
System Safety	Reduction in the overall safety margin of pipeline systems; may lead to leakage or rupture, causing cascading structural failures	Welding defects produce cumulative effects on system safety; local performance degradation leads to stress redistribution, increasing the load on other joints or components and raising the risk of progressive failure	Accident analyses of natural gas pipeline networks; structural integrity assessment of multi-joint pipeline systems

**Table 2:** Typical Welding Defects in the Fusion Zone.

Defect Type	Analysis Dimension	Detailed Description	Main Causes	Detectability
Cold Welding	Formation Mechanism	The fusion temperature fails to reach the material melting point, or the heating time is insufficient, resulting in inadequate molecular chain diffusion and entanglement	Insufficient temperature; inadequate heating time; non-uniform heat input	High (not visible)
	Typical Characteristics	Unbonded interface, often manifested as weak or incomplete bonding at the joint interface		
	Consequences	Significant reduction in joint strength and toughness; acts as a crack initiation site and has been widely validated as a critical defect in safety assessments		
Over-welding	Formation Mechanism	Excessive heat input or prolonged heating time leads to thermal degradation of polyethylene materials	Excessive heat input; prolonged heating time; lack of environmental temperature compensation	Medium (partially visible)
	Typical Characteristics	Melt overflow, bubble formation, and degradation of polymer chains		
	Consequences	Degradation of local material properties, increased stress concentration, and higher risk of failure		
Voids	Formation Mechanism	Gas entrapment during welding, or the presence of moisture and impurities in materials; gas cannot escape during melting, forming microvoids [48]	Gas entrapment; material moisture; impurities	Medium (requires NDT)
	Typical Characteristics	Microvoids within the weld, reducing structural compactness		
	Consequences	Reduced density and crack resistance of the weld		
Structural Deformity	Formation Mechanism	Improper alignment, asymmetric heating, or pipe misalignment during welding	Poor alignment; asymmetric heating; pipe misalignment	Low (visible)
	Typical Characteristics	Asymmetric or distorted weld structure		
	Consequences	Under service loading, uneven stress distribution occurs, reducing overall structural stability [49]		

**Table 3:** Comparison of Major Inspection Technologies for Electrofusion Joints.

Inspection Technique	Primary Function	Advantages	Limitations
Conventional Ultrasonic Testing (UT)	Detection of internal defects	Relatively low cost and strong field applicability	Moderate resolution and strong dependence on operator experience
Phased Array Ultrasonic Testing (PAUT)	Defect localization and imaging	High accuracy and strong visualization capability	Expensive equipment and high operational requirements
Infrared Thermography (IRT)	Monitoring of welding temperature fields	Non-contact and capable of real-time monitoring	Difficult to identify internal defects
Radiographic Testing (RT)	Detection of internal structural abnormalities	Intuitive imaging and effective void detection	Radiation hazards and relatively low efficiency
Industrial Computed Tomography (CT)	Three-dimensional analysis of internal structures	Highest accuracy and comprehensive results	High cost and slow inspection speed
Visual Inspection	Examination of surface quality	Simple, rapid, and low cost	Limited to surface defects only
Mechanical Testing	Verification of joint performance	Direct and reliable results	Destructive to specimens and unsuitable for full inspection
AI-Based Intelligent Diagnosis	Automatic defect recognition	High efficiency and good consistency	Strong dependence on data training

**Table 4:** Development Trends of Electrofusion Welding for Polymeric Pipelines.

Research Theme	Description	Research Methods/Techniques
Early Understanding	Assumes a simplified heat input–cooling process based on linear heat conduction models	Basic heat conduction calculations
Current Understanding	Thermo-mechanical response of materials during welding significantly influences joint formation and performance	Thermo-mechanical coupled numerical simulation
Effect of Heat Input	Excessive heat input may not only lead to over-welding but also cause local thermal degradation of polyethylene materials	Experimental analysis of thermal degradation behavior
Material Response	Nonlinear material behavior under thermo-mechanical coupling determines weld quality	Multi-physics coupled simulation
Heat-Affected Zone (HAZ)	The width of the heat-affected zone shows a nonlinear relationship with welding time and heat input	Microstructural characterization of HAZ
Crystallinity Evolution	Crystallinity changes exhibit a nonlinear relationship with welding time and heat input	Crystallinity characterization (XRD, DSC, etc.)
Key Findings	The interaction between thermal fields and material response is complex and requires in-depth analysis	Integration of experimental studies and theoretical modeling
Engineering Implications	Provides a theoretical basis for precise welding process control and optimization of joint performance	Process parameter optimization and online monitoring [77]

## Data Availability

The original contributions presented in this study are included in the article. Further inquiries can be directed to the corresponding authors.
